# Primary solitary fibrous tumors of liver: a case report and literature review

**DOI:** 10.1186/1746-1596-8-195

**Published:** 2013-12-02

**Authors:** Qiang Liu, Jing Liu, Wenyou Chen, Shunbao Mao, Yihe Guo

**Affiliations:** 1 Department of General Surgery, The 175th Hospital PLA (Affiliated Dongnan Hospital of Xiamen University), NO. 269, Zhanghua Middle Road, Zhangzhou 363000, Fujian Province, China; 2 Department of Pathology, the 175th Hospital of PLA (Southeast Hospital Affiliated to Xiamen University), NO. 269, Zhanghua Middle Road, Zhangzhou 363000, Fujian Province, China

**Keywords:** Solitary fibrous tumors, Liver, Pathology

## Abstract

A 42-year-old male presented right upper abdomen pain for more than 6 days, which misdiagnose calculus of intrahepatic duct and acute cholecystitis. An approximately 1.5 cm x 1.0 cm x 1.0 cm nodule was found and resected in left lateral lobe of hepatic. Pathological examination showed spindle cell and fibroblast -like cells within the collagenous stroma. Immunohistochemically, these spindle tumor cells showed diffuse Vim and Bcl-2 positive reactivity, but S-100 protein and HMB45 were negative. The post-operative course was uneventful. Solitary fibrous tumors of the liver, although rare, should be differentiated from mesenchymal lesions of the liver.

**Virtual slide:**

The virtual slide(s) for this article can be found here: http://www.diagnosticpathology.diagnomx.eu/vs/4214341041091088.

## Introduction

Solitary fibrous tumors (SFTs) was first differentiated from mesothelioma by Klemperer and Rabin in 1931 [1], which is an uncommon neoplasm of mesenchymal origin that primarily affects the pleura and mediastinum. SFTs may occur elsewhere in the body including respiratory tract, peritoneum or mesentery, eyes, breasts and central nervous system and other soft tissues. while liver parenchyma is a rare location of SFT, with only 42 cases reported in the literature. Preoperative diagnosis for primary SFTs of liver is difficult because of atypical symptom, and it mainly depends on postoperative pathological examination. Because four malignant transformation or metastasis cases has been reported, the best surgical treatment is a complete resection including the edge of the tumor, and Long-term follow-up is recommended. We herein report a case of primary solitary fibrous tumor of liver and review the previous reported cases, then discuss the possible differential diagnosis.

### Case report

**Figure 1:**
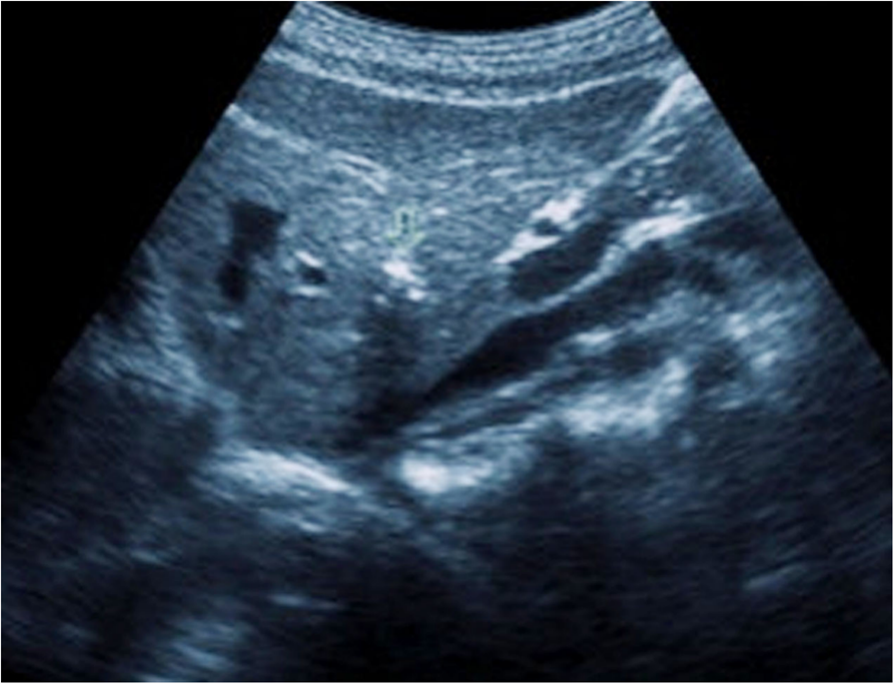
Ultrasonography (US) revealed calculus of intrahepatic duct.

**Figure 2:**
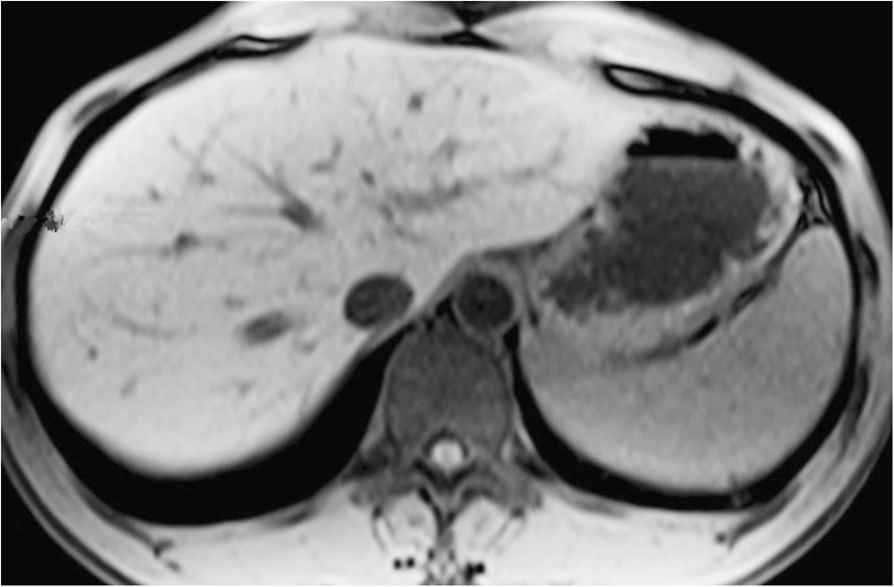
Magnetic resonance cholangiopancreatography(MRCP) revealed Gallbladdercalculi and cholecystitis and calculus of intrahepatic duct.

A 42-year-old male was admitted to the Department of General Surgery with right upper abdomen pain for more than 6 days. There was no history of vomiting, fever, chill, jaundice and gastrointestinal bleeding. He denied any history of surgery, blood transfusion, alcohol abuse or medication. His vital signs (heart rate, blood pressure, respiratory rate and body temperature) were stable. Physical examination was unremarkable except Murphy(+). Laboratory date were normal, except the white blood cell (WBC) count was 13.79 × 10^9^. Ultrasonography (US) revealed calculus of intrahepatic duct (Figure 1). Magnetic resonance cholangiopancreatography(MRCP) revealed Gallbladder calculi, cholecystitis and calculus of intrahepatic duct (Figure 2). Based on the above examinations, the preoperative diagnosis of calculus of intrahepatic duct and acute cholecystitis was given.

**Figure 3:**
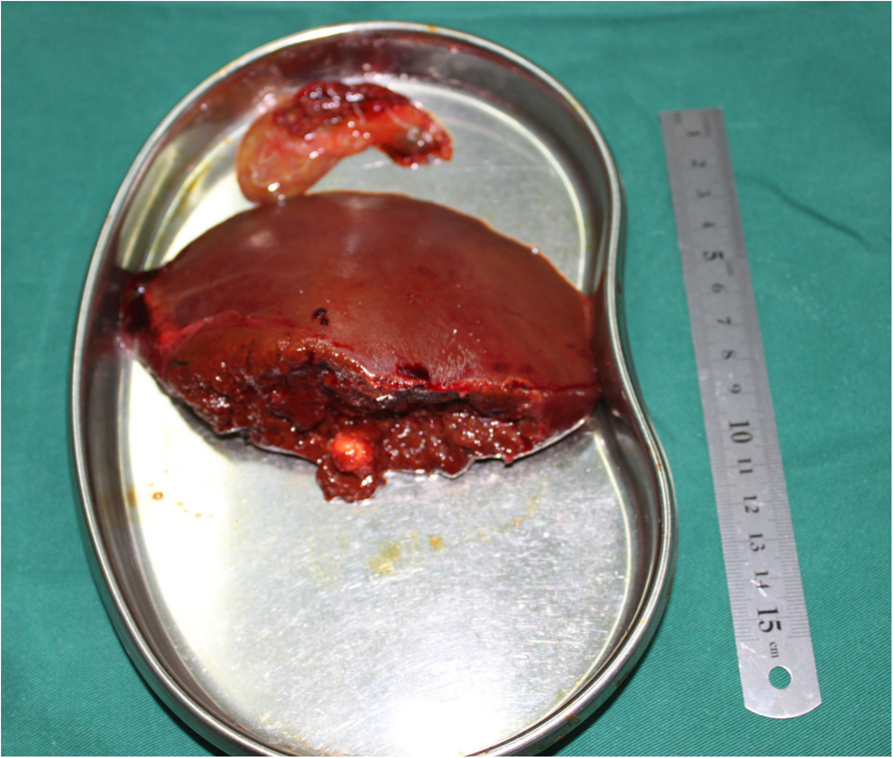
Gross appearance of the resected specimen, which measured 1.5 cm × 1.0 cm × 1.0 cm in left lateral lobe of hepatic.

**Figure 4:**
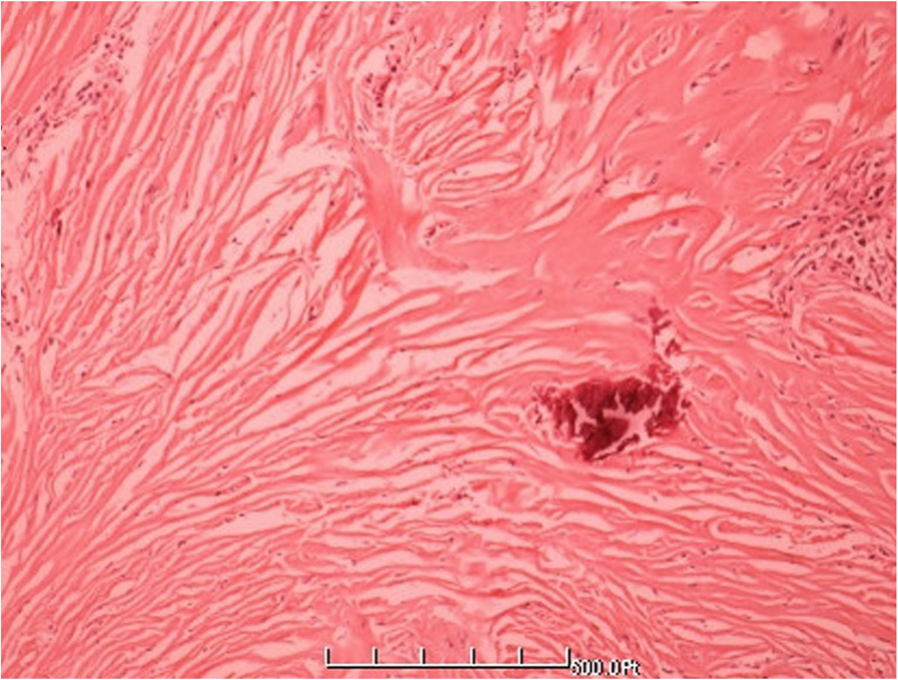
**Histologic features of the lesion showed the tumor was composed of small spindle cells, variably admixed with fiber texture (HE** **×** **100).**

**Figure 5:**
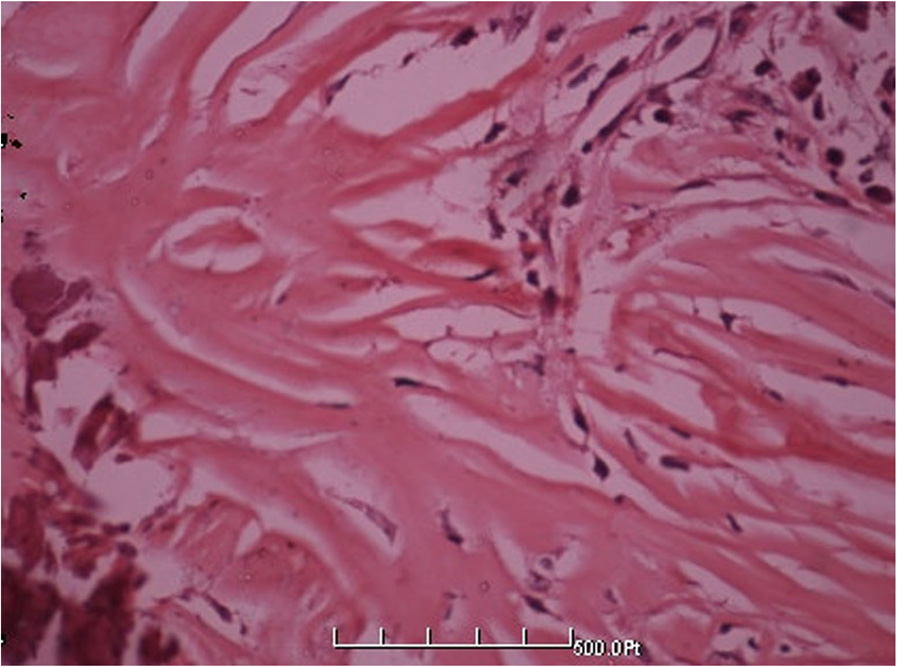
**Higher magnification showing fiber texture and spindle cells list storiform (HE** **×** **400).**

**Figure 6:**
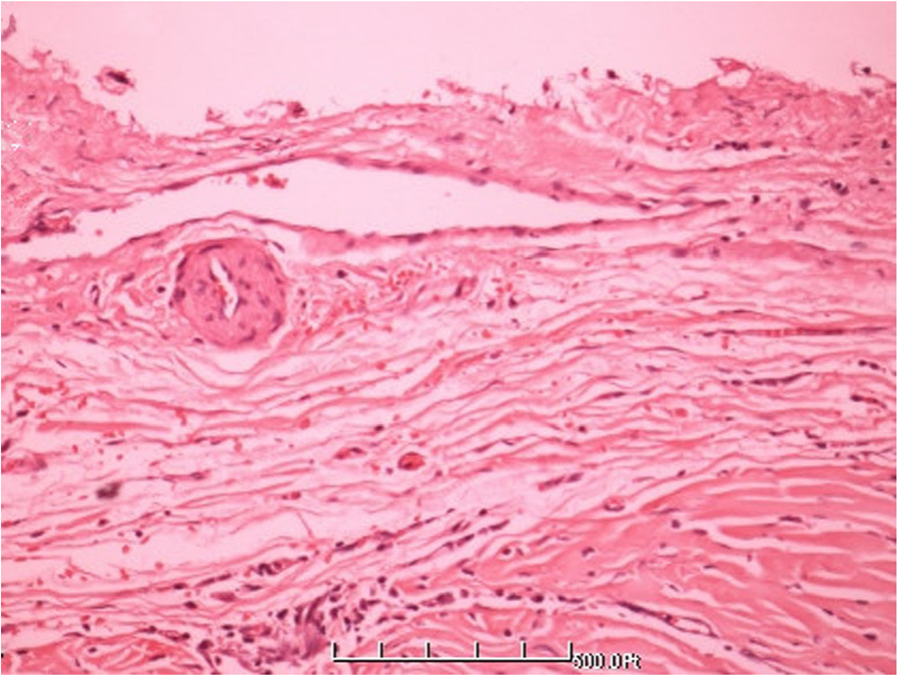
A clear demarcation between the tumor edge area and normal liver tissue (HE × 200).

**Figure 7:**
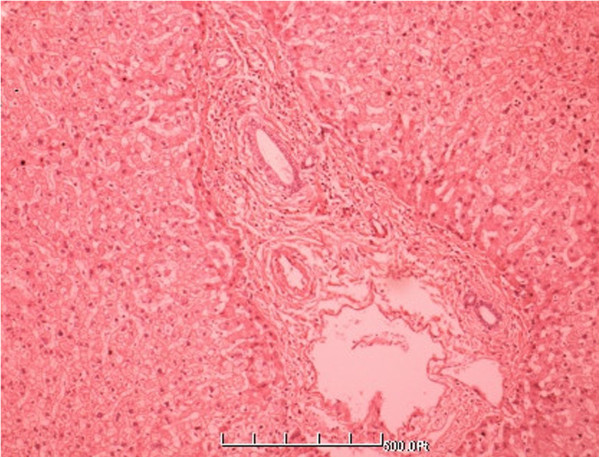
**The distribution of typical blood vessel in the normal liver tissue (HE** **×** **200).**

**Figure 8:**
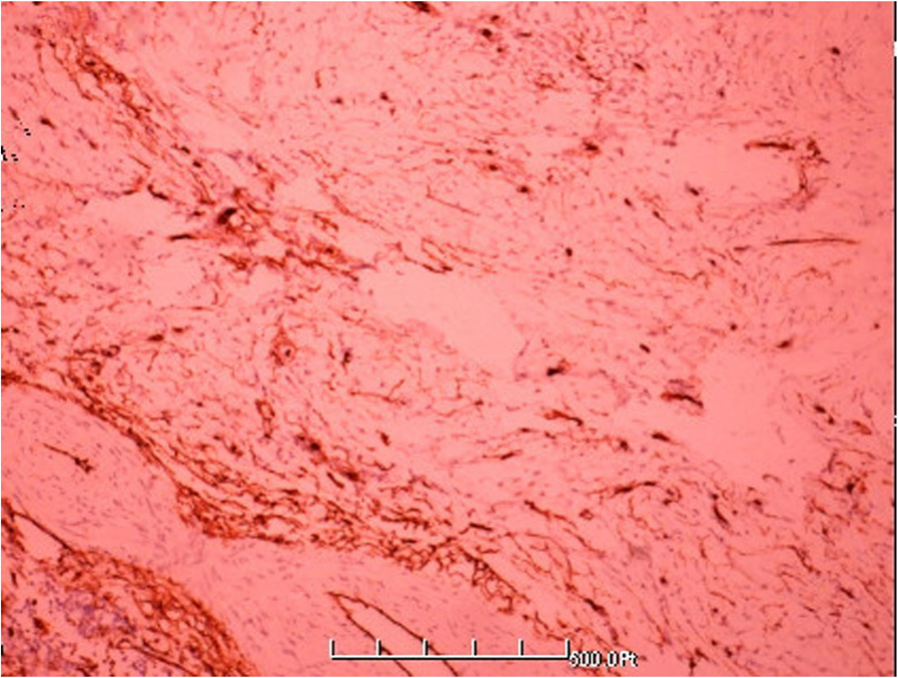
Tumor cells showing diffuse immunohistochemical positivity for CD-34.

**Figure 9:**
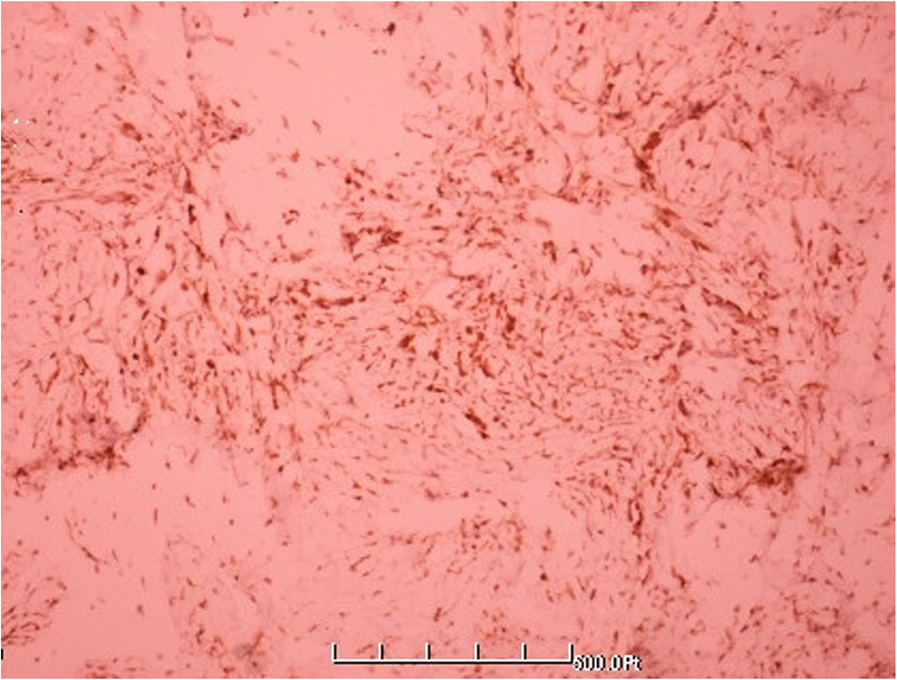
Tumor cells showing diffuse immunohistochemical positivity for Bcl-2.

At laparotomy, the gallbladder was slightly swollen and high tension, which is 10 cm × 6 cm × 3 cm, especially gall bladder wall. An approximately 1.5 cm × 1.0 cm × 1.0 cm nodule was found in left lateral lobe of hepatic (Figure 3), so resection of the gallbladder and left lateral lobe of hepatic was performed. pathological examination of the resected specimen showed spindle cell and fibroblast -like cells within the collagenous stroma (Figures 4 and 5). Moreover, A clear demarcation was found between the tumor edge area and normal liver tissue (Figure 6), and the distribution of typical blood vessel in the normal liver tissue (Figure 7). Immunohistochemically, these spindle tumor cells showed diffuse CD34 and Bcl-2 positive reactivity (Figures 8 and 9), S-100 protein and HMB45 were negative, Masson colouration disclosed lots of collagenous fiber. Postoperative course was uneventful, with hospital discharge at the eleventh day. Although recurrence and metastasis was not seen, we will pay attention to long-term follow-up.

## Discussion

Solitary fibrous tumors (SFTs) is an uncommon neoplasm of mesenchymal origin that primarily affects the pleura and mediastinum. SFTs may occur elsewhere in the body including respiratory tract, peritoneum or mesentery, eyes, breasts and central nervous system and other soft tissues. There are only 42 reported cases of SFTs originating from the liver [2-35], and four of them were malignant (Tables 1 and 2) [32-35].

**Table 1:** Clinical data of liver SFT in 42 patients previous reported in the literature

**No**	**Author**	**Year**	**Age/gender**	**Treatment**	**Lobe**	**IH**	**Follow-up**
1	Edmondson	1958	16/F	Excision	R	na	24 m
2	Edmondson	1958	na	Excision	R	na	na
3	Nevius	1959	56/M	Radiation	R	na	2d
4	Ishak	1976	62/M	Excision	L	na	na
5	Ishak	1976	62/F	Excision	L	na	na
6	Kim	1983	27/F	Excision	L	na	6 m
7	Kottke-Marchant	1989	84/F	Excision	L	na	53 m
8	Barnoud	1996	50/M	Excision	R	CD34+	38 m
9	Levine	1997	57/M	Excision	L	CD34+	na
10	Guglielmi	1998	61/F	Excision	R	CD34+	72 m
11	Moran	1998	62/F	Excision	na	CD34+	na
12	Moran	1998	34/F	no	na	na	Autopsy
13	Moran	1998	57/F	Excision	na	CD34+	na
14	Moran	1998	32/M	Excision	na	CD34+	na
15	Moran	1998	68/F	Excision	na	CD34+	2d
16	Moran	1998	83/F	Excision	R	CD34+	6d
17	Moran	1998	72/F	Excision	L	CD34+	12 m
18	Moran	1998	62/F	Excision	L	CD34+	na
19	Moran	1998	50/F	Excision	L	CD34+	na
20	Fuksbrumer	2000	40/F	Excision	R	CD34+	na
21	Fuksbrumer	2000	71/F	Excision	R	CD34+	na
22	Fuksbrumer	2000	80/M	Excision	R	CD34+	na
23	Yilmaz^#^	2000	25/F	Excision	R	Vimentin+	6 m
24	Lin	2001	75/M	Excision	R	CD34+	11 m
25	Saint-Marc	2002	69/F	Excision	R	CD34+	15 m
26	Neeff	2004	63/F	Excision	L	CD34+	11 m
27	Chithriki	2004	75/F	Excision	R	CD34+	6 m
28	Venarecci	2005	65/F	Excision	R	CD34+	30 m
29	Ji	2006	46/F	Excision	R	CD34+	na
30	Lehmann	2006	63/F	Excision	R	CD34+	96 m
31	Nath	2006	65/F	Excision	R	CD34+	10 m
32	Terkivatan	2006	74/M	Excision	L	CD34+	12 m
33-35	Weitz	2007	na	Excision	na	na	na
36	Obuz	2007	52/M	Excision	L	CD34+	22 m
37	Perini	2007	40/F	Excision	L	CD34+	49 m
38	Chan^#^	2007	70/M	Excision	R	CD34+	12 m
39	F. Famà	2007	68/M	Excision	R	CD34+	25 m
40	Ka-Jeong Kim	2009	71/F	Excision	L	CD34+	na
41	Brochard^#^	2010	54/M	Excision	R	CD34+	72 m
42	Peng L^#^	2011	24/F	Excision	R	CD34+	16 m

^#^malignant liver SFT; F, female; M, male; No, not operated; R, right; L, left; Na, not available; d, days; m, months; IH, Immunohistochemistry.

**Table 2:** Summary of the 42 cases of liver SFT

**Items**	**Summarized date**
Sex	26 female, 12 male, 4 na
Age(y)	Range 16-84, average 52
Lobe	21R,13 L, 8 na
Treatment	40 excision, 1 radiation,1 no
Survial	Range2d-96 m, average 48 m
Malignant	4 in 42 cases

F, female; M, male; No, not operated; R, right; L, left; Na, not available; Ms, months; IH, Immunohistochemistry.

SFTs is a form of borderline tumors, without potential predisposing factors. SFT of the liver is more common in women (26 cases were female in 42 patients), and typically affects middle-aged adults (mean age 52, range 16–84) and it slowly grows with the performance of local asymptomatic or abdominal pain, but occasional cases have presented with hypoglycaemia. The corresponding clinical symptoms appearance only when the tumor grows to a certain size, or when vital structures are involved. Although there are no definitive criteria of malignancy for SFT, About 10% SFTs are malignant or potentially malignant. The current World Health Organization (WHO) classification criteria of soft tissue tumors is used to identify malignant SFT, these criteria include a large tumor size (more than 5 or 10 cm), a sessile lesion, infiltrative margins, hypercellularity, nuclear pleomorphism, an area of tissue necrosis or hemorrhage and an increased mitotic index (more than 4 mitoses in 10 HPFs) [35]. The patient suffered right upper abdominal pain because of calculus of intrahepatic duct, Gallbladder calculi, cholecystitis, and a nodule was found in left lateral lobe of hepatic at laparotomy, which led to the misdiagnose of calculus of intrahepatic duct causing it. We resected the gallbladder and placeholder nodules, postoperative pathology diagnosed resected specimen as the occupying nodules solitary fibrous tumor.

Iconography examinations neither specifically diagnose SFTs nor identify the character of it, benign or malignant. Generally speaking, if CT images show limit isolated hyper vascular abnormal parceled tissue, we will suspected the possibility of SFTs. The SFTs present low signal in MRI scans because of mature fibrous tissue T2WI in tumor [36]. In order to confirm the diagnosis and assessment before operative, some scholars have suggested percutaneous liver biopsy guided by radiation. However, because this invasive procedures are only conducted on tumor biopsy or part of the edge specimens, if the tumor proliferates, it may be missed or misdiagnosed. In addition, due to its unclear nature, this procedure may cause the risk of tumors growing along the puncture path [37]. Therefore, for the diagnosis and assessment of SFTs, percutaneous liver biopsy is not recommended.

Although imaging examination will certainly help for SFTs, but diagnosis of SFTs mostly depend on postoperative pathological histological features and immunohistochemical evaluation. General histological observation can find that bland spindle cells arranged in a “patternless” pattern, with alternating hypocellular and hypercellular areas, interspersed with thick, “keloidal” collagen bundles. A haemangiopericytoma-like vascular pattern is a common feature. Typical indicators of immunohistochemical revealed that CD34 and vimentin are strongly positive, So is Bcl-2, which differentiate from other tumors [14].

SFT should be differentiated from some other haemangiopericytoma-like tumors: Haemangiopericytoma (HPC), synovial sarcoma, peripheral nerve sheath tumor (PNST). HPC is most likely to be confused with SFT in some other haemangiopericytoma-like tumors. HPC is composed of spindle or oval shaped cells with scanty cytoplasm and plump nucleus, which arrange closely. The most important feature in HPC is extensive irregular staghorn vascular pattern, Immunohistochemistry tumor cells showed vimentin and actin focal positivity and were negative for CD34 [38]. Other haemangiopericytoma-like soft tissue tumors what should be distinguished from SFT is synovial sarcoma. It is a high-grade biphasic that characterized by epithelioid and spindle cells with a solid growth pattern, Areas of myxoid, neural, necrosis and a vasculature with numerous dilated vessels resembling hemangiopericytoma were observed. what is more, Immunohistochemical studies for EMA and CK showed strong positivity, The fusion gene SYT-SSX and translocation t(X; 18) are the special marker for synovial sarcoma, which is not seen in any other spindle cell tumors [39]. PNST is another rare haemangiopericytoma-like soft tissue tumours that may be confused with SFT. We can see the alternating hypercellular area and hypocellular area, the spindle cells form Wagner-Meissner structure, which makes the spindle cell with wavy or pleomorphic nuclei and elongated cytoplasmic. Intercellular substance has many blood vessels, just as haemangiopericytoma-like formation. PNST is easy to become malignant tumour, which high cellularity, nuclear atypia, and increased mitotic rate, necrosis, and endothelial proliferation can be found [40]. Immunohistochemically, the tumor cells were focally positive for S-100 protein, but negative for desmin, CD34 and EMA.

There are only 42 reported cases of SFTs originating from the liver, but it should be differentiated from some other tumours originating from the liver. Among the primary neoplasms of the liver that can be confuse with SFTs are Leiomyoma, Hepatic metastatic gastrointestinal stromal tumors (GISTs), Hepatic angiomyolipoma (AML), Sarcoma hepatocellular carcinoma (HCC) and Inflammatory myofibroblastic tumour of the liver. Leiomyoma of liver is a very rare tumor in liver. It consists of intersecting bundles of smooth muscle cells with cigarshaped nuclei that express MSA and SMA but not CD34 [14]. Gastrointestinal stromal tumors (GISTs) have a plentiful histomorphometric, and liver is the most common metastatic part. Most hepatic metastatic GISTs is composed of spindle and/or predominantly epithelioid cells with round to oval-shaped nuclei and eosinophilic cytoplasm. We even can see that the tumor cells proliferated in a diffuse or sheetlike pattern with focal myxoid stroma and microcystic change. About 95% are positive for KIT (CD117), 60–70% for CD34, 30–40% for smooth muscle actin, while cytokeratins and EMA are negative [41]. Angiomyolipoma is commonly seen in the kidneys, but infrequently in liver. These tumours are characterized by co-expression of smooth muscle and melanocytic markers. Microscopically, the tumours are composed of admixture of adipose tissue, smooth muscle and blood vessels, The histological patterns described include lipomatous, myomatous, angiomatous, trabecular, pelioid, inflammatory and mixed pattern, and the cells were positive for HMB-45. Sarcoma hepatocellular carcinoma can be easily distinguished from SFT by trabecular hepatocellular and sarcoma composition, Immunohistochemistry demonstrates positivity for CK. Inflammatory myofibroblastic tumour of the liver consists of compact spindle cells (myofibroblasts, fibroblasts) intermingled with inflammatory cells, predominantly plasma cells, lymphocytes, as well as eosinophils. Immunohistochemically, the tumor cells demonstrate positivity for vimentin, MSA, SMA and cytokeratin [42].

Give that SFTs is a rare soft tissue tumor and occurs most commonly in the pleura, there is need to make differential diagnosis of SFTs in relationship to the pleura, such as chronic organized pneumonia, pneumoconiosis, pleural mesothelioma and some other uncommon pulmonary tumor. Chronic organized pneumonia is the most common disease of the lung and can be easily differentiated from SFTs by its clinical presentation and typical inflammatory exudation in pulmonary alveolus cavity. Pneumoconiosis, including silicosis and asbestosis of the lung, associated with occupational exposure to silica and asbestos occur across a broad range of industries. Silicosis is characterized by typical silicotic nodule and diffuse pulmonary fibrosis [43], and asbestosis is typical asbestos corpuscle, pleural plaques, diffuse pleural thickening and pulmonary fibrosis [44], which helps distinguish pneumoconiosis from SFTs of lung. Pleural mesothelioma, mostly malignant, is associated with the devolpment of pneumoconiosis. Malignant pleural mesothelioma (MPM) is divided into three major histological sub-types: sarcomatoid biphasic and epithelioid. Diffuse pleural thickening with multiple nodular can be found and several immunohistochemical panels are proposed to distinguish between MPM and SFTs, such as strong positive for calretinin and other useful antibodies include thrombomodulin, mesothelin and cytokeratin 5 [45]. In addition, the differential diagnosis should also include some uncommon pulmonary tumor, such as Inflammatory myofibroblastic tumor (IMT) of lung, pulmonary sclerosing hemangioma (PSH) and clear cell “sugar” tumor (CCST) of lung. IMT of lung is a rare benign mesenchyma lesion which is closely related to recurrent respiratory infections, Histologically, it shows the admixture of spindle-shaped and ovoid cells with a prominent inflammatory infiltrate [46]. PSH is usually easily diagnosed based on the typical architectural patterns including papillary, sclerotic, solid, hemorrhagic pattern and the cuboidal cells or polygonal cells types [47]. CCST of the lung is typically diagnosed by computed tomography–guided transthoracic fine-needle aspiration biopsy and core-needle biopsy, And cytologic features is large irregular clusters of bland-appearing polygonal and spindle-shaped cells with vacuolated granular cytoplasm [48]. Besides, differential diagnosis of SFTs in abdominal cavity is gastrointestinal stromal tumors (GISTs) which we have elaborated in preceding paragraph. And the differential diagnosis of SFTs in soft tissue of shoulder and back neck is spindle cell lipoma which is composed of spindle cells, mature adipose tissue and varying amounts of collagen fibers [49]. In additional, the SFTs of the hub, orbit and paranasal sinuses should be differentiated from fibroblast cell meningioma, from which we can observe island meningeal skin cells and grit in the spindle-shaped beam of tumor cells, and it can also be ruled out by positive immunohistochemical staining for EMA, CK and S-100 and negative staining for CD34 [50].

It was reported that a few non-islet cell tumor hypoglycemia syndrome (NICTH) is associated with the liver fibroma [51]. It has been foud that NICTH syndrome is related with the excess IGF-II (insulin-like growth factor-II) tumor produces. Clinically that overproduction of IGF-II by solitary fibrous tumor of liver led NICTH to be rarely reported, and the concrete mechanism needs further study. But it remind us that for those patients with persistent low blood sugar but with normal islet cells for unknown reasons, if liver occupies, solitary fibrous tumor should be considered.

## Conclusion

There is no clear diagnostic criteria for SFT of liver, it should be made by their microscopical and immunohistochemical features. SFT of liver should be considered in the differenial diagnosis of lesions with Haemangiopericytoma, synovial sarcoma, peripheral nerve sheath tumor and some other tumours originating from the liver.

### Consent

Written informed consent was obtained from patient for publication of this case report and any accompanying images.

## Abbreviations

SFTs: Solitary fibrous tumours; HPC: Haemangiopericytoma; PNST: Peripheral nerve sheath tumor; GISTs: Gastrointestinal stromal tumors; AML: Angiomyolipoma; HCC: Hepatocellular carcinoma; IMT: Inflammatory myofibroblastic tumor; PSH: Pulmonary sclerosing hemangioma; CCST: Clear cell “sugar” tumor; NICTH: Non-islet cell tumor hypoglycemia syndrome.

## Competing interests

The author’s declared no potential competing interest with respect to the research, authorship, and/or publication of this article.

## Authors’ contributions

QL, WYC and YHG performed the histological examination of the tumor and were major contributors to the writing of the manuscript. JL and SBM are the surgeons who operated on the patient and interpreted the patient data. All authors read and approved the final manuscript.
